# Effect of Intraoperative Esketamine Infusion on Postoperative Sleep Disturbance After Gynecological Laparoscopy: A Randomized Clinical Trial

**DOI:** 10.1001/jamanetworkopen.2022.44514

**Published:** 2022-12-01

**Authors:** Di Qiu, Xing-Ming Wang, Jin-Jin Yang, Sai Chen, Cai-Bao Yue, Kenji Hashimoto, Jian-Jun Yang

**Affiliations:** 1 Department of Anesthesiology, Pain and Perioperative Medicine, The First Affiliated Hospital of Zhengzhou University, Zhengzhou, Henan, China; 2 Division of Clinical Neuroscience, Chiba University Center for Forensic Mental Health, Chiba, Japan

## Abstract

**Importance:**

Postoperative sleep disturbance (PSD) is common in patients after surgery.

**Objective:**

To examine the effect of intraoperative esketamine infusion on the incidence of PSD in patients who underwent gynecological laparoscopic surgery.

**Design, Setting, and Participants:**

This single-center, double-blind, placebo-controlled randomized clinical trial was conducted from August 2021 to April 2022 in the First Affiliated Hospital of Zhengzhou University in China. Participants included patients aged 18 to 65 years with an American Society of Anesthesiologist Physical Status classification of I to III (with I indicating a healthy patient, II a patient with mild systemic disease, and III a patient with severe systemic disease) who underwent gynecological laparoscopic surgery. Patients were randomly assigned to either the esketamine group or control group. Data were analyzed using the per protocol principle.

**Interventions:**

Patients in the esketamine group received a continuous infusion of esketamine, 0.3 mg/kg/h, intraoperatively. Patients in the control group received an equivalent volume of saline.

**Main Outcomes and Measures:**

The primary outcome was the incidence of PSD on postoperative days (PODs) 1 and 3. Postoperative sleep disturbance was defined as a numeric rating scale score of 6 or higher or an Athens Insomnia Scale score of 6 points or higher. The secondary outcomes included postoperative anxiety and depression scores using the Hospital Anxiety and Depression Scale, postoperative pain using the visual analog scale, postoperative hydromorphone consumption, and risk factors associated with PSD.

**Results:**

A total of 183 female patients were randomized to the control group (n = 91; median [IQR] age, 45 [35-49] years) and the esketamine group (n = 92; median [IQR] age, 43 [32-49] years). The incidence of PSD in the esketamine group was significantly lower than in the control group on POD 1 (22.8% vs 44.0%; odds ratio [OR], 0.38 [95% CI, 0.20-0.72]; *P* = .002) and POD 3 (7.6% vs 19.8%; OR, 0.33 [95% CI, 0.13-0.84]; *P* = .02). There were no differences in postoperative depression and anxiety scores between the 2 groups. Postoperative hydromorphone consumption in the first 24 hours (3.0 [range, 2.8-3.3] mg vs 3.2 [range, 2.9-3.4] mg; *P* = .04) and pain scores on movement (3 [3-4] vs 4 [3-5] points; *P* < .001) were significantly lower in the esketamine group than in the control group. On multivariable logistic regression, preoperative depression (OR, 1.31; 95% CI, 1.01-1.70) and anxiety (OR, 1.67; 95% CI, 1.04-1.80) scores, duration of anesthesia (OR, 1.04; 95% CI, 1.00-1.08), and postoperative pain score (OR, 1.92; 95% CI, 1.24-2.96) were identified as risk factors associated with PSD.

**Conclusions and Relevance:**

Results of this trial showed the prophylactic effect of intraoperative esketamine infusion on the incidence of PSD in patients who underwent gynecological laparoscopic surgery. Further studies are needed to confirm these results.

**Trial Registration:**

Chinese Clinical Trial Registry Identifier: ChiCTR2100048587

## Key Points

**Question:**

Does intravenous infusion of esketamine prevent postoperative sleep disturbance (PSD) at days 1 and 3 after gynecological laparoscopic surgery?

**Findings:**

In this randomized clinical trial of 183 female patients who underwent gynecological laparoscopic surgery in China, esketamine significantly decreased the incidence of PSD.

**Meaning:**

Findings of this trial indicate that intraoperative esketamine infusion had a prophylactic effect on the incidence of PSD in patients who underwent gynecological laparoscopic surgery; further studies are needed to confirm these findings.

## Teaser

This randomized clinical trial examines the effect of intraoperative esketamine infusion on incidence of sleep disturbance following gynecological laparoscopic surgery.

## Introduction

Postoperative sleep disturbance (PSD), which can be in the form of sleep deprivation, circadian disruption, and abnormal architecture, occurs in 15% to 72% of patients after surgery.^1,2,3^ It is associated with several factors, including older age, female sex, postoperative pain and anxiety, postoperative nausea and vomiting, and maladaptation to the ward environment. Postoperative sleep disturbance could lead to postoperative delirium and cognitive impairment, exacerbate acute postoperative pain, and delay postoperative recovery.^2,4,5,6,7^ Pharmacological interventions, such as short-acting non-benzodiazepine zolpidem and melatonin, have often been used to improve postoperative sleep quality.^8^ However, to our knowledge, there are no studies regarding prophylactic agents for PSD.

(*R*,*S*)-ketamine, an *N*-methyl-D-aspartate receptor (NMDAR) antagonist, is a racemic mixture of equal amounts of (*R*)-ketamine (ie, arketamine) and (*S*)-ketamine (ie, esketamine), with esketamine having a greater affinity for the NMDAR.^9,10^ A subanesthetic dose of (*R*,*S*)-ketamine (0.5 mg/kg, 40-minute intravenous [IV] infusion) is known to produce rapid-acting and sustained antidepressant actions in patients with major depressive disorder (MDD) resistant to treatment.^11,12^ Esketamine is used for anesthesia in some countries, including China. Subanesthetic doses (0.2 and 0.4 mg/kg, IV infusion) of esketamine have demonstrated rapid and robust antidepressant efficacy in patients with treatment-resistant MDD, although it had transient psychotomimetic and dissociative adverse effects.^13^ In 2019, an esketamine nasal spray (Johnson & Johnson) was approved in the US and Europe for treatment-resistant depression.

Postoperative sleep disturbance is often seen alongside depression in patients after surgery.^14,15,16,17^ In addition to its antidepressant actions, (*R,S*)-ketamine infusion has been demonstrated to improve sleep disturbance in patients with MDD and sleeping problems, suggesting the potential of (*R,S*)-ketamine for sleep disturbance.^18,19,20^ It has been suggested that the antidepressant effect of (*R,S*)-ketamine is connected with the neurobiological structure of the wake, sleep, and circadian rhythms.^21^ However, it is unknown whether IV infusions of low-dose esketamine intraoperatively can reduce the incidence of PSD. This trial aimed to examine the effect of intraoperative esketamine infusion on PSD in patients who underwent gynecological laparoscopic surgery.

## Methods

### Study Design

This prospective, double-blind, placebo-controlled randomized clinical trial was approved by the Institutional Scientific Research and Clinical Trials Ethics Committee of the First Affiliated Hospital of Zhengzhou University. The trial protocol is provided in Supplement 1. The protocol was explained to patients before the trial started, and written informed consent was obtained from all participants. We followed the Consolidated Standards of Reporting Trials (CONSORT) reporting guideline.

The trial enrolled patients who were aged 18 to 65 years, had an American Society of Anesthesiologist (ASA) Physical Status classification of I to III (I indicates a healthy patient, II indicates a patient with mild systemic disease, and III indicates a patient with severe systemic disease), and underwent gynecological laparoscopic surgery from August 2021 to April 2022 in the First Affiliated Hospital of Zhengzhou University in China. The exclusion criteria were as follows: (1) patient refusal to participate in the study, (2) body mass index (calculated as weight in kilograms divided by height in meters squared) higher than 30, (3) preoperative Pittsburgh Sleep Quality Index (PSQI) higher than 7, (4) recent history of drug abuse, (5) contraindications or allergy to esketamine, (6) cognitive dysfunction or inability to communicate, and (7) inability to use a patient-controlled IV analgesia pump. Race and ethnicity data were not collected because all trial participants were Han Chinese.

**Figure 1.**
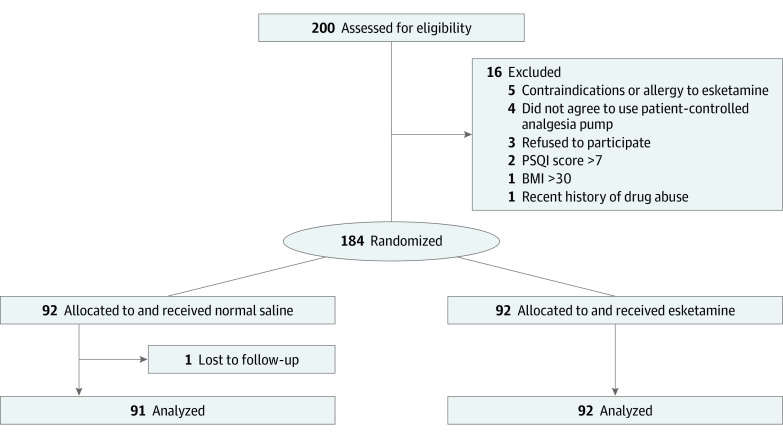
Participant Flowchart

Patients were randomly assigned in a 1:1 ratio to receive either esketamine infusion, 0.3 mg/kg/h (Jiangsu Hengrui Pharmaceutical Co Ltd), or an equivalent volume of saline (Figure 1) using a computer-generated randomization table. For allocation concealment, group assignments were placed in sealed envelopes, which were sequentially handed to a nurse who was not involved in the study. Both esketamine and saline were prepared in identical 20-mL syringes by the same nurse. Anesthesiologists (D.Q., S.C., Jin-Jin Y.) who were involved in patient management and had participated in the postoperative follow-up were blinded to the group assignment. Esketamine, 50 mg, was diluted to a total of 20 mL with saline solution. Group assignments were not revealed until patients were discharged from the hospital.

### Anesthesia and Postoperative Analgesia Management

Routine intraoperative monitoring was established, including pulse oximetry, electrocardiography, and noninvasive blood pressure measurement. Anesthesia was induced with etomidate, 0.2 mg/kg; alfentanil, 40-50 μg/kg; and then rocuronium, 0.9 mg/kg, to facilitate the insertion of a laryngeal mask airway (AuraGain; Ambu). Anesthesia was maintained with remifentanil, 0.1-0.2 μg/kg/min, and 2% sevoflurane in 50% oxygen, 2 L/min, and the remifentanil infusion rate was titrated to keep the heart rate and arterial blood pressure within 20% of baseline. Palonosetron, 0.25 mg, was intravenously administered at the end of the surgery to prevent postoperative nausea and vomiting. After completing surgery, the laryngeal mask was pulled out when adequate muscle strength was established. Postoperative pain was managed using hydromorphone, 0.2 mg/kg in 200 mL saline, by a patient-controlled IV analgesia pump: background infusion 2 mL/h for 48 hours, bolus 2 mL, lockout time 10 minutes. Flurbiprofen axetil, 50 mg, was intravenously administered as a rescue analgesic if the pain score was greater than 4.

### Outcome Measurements

Postoperative sleep quality was evaluated using the numeric rating scale (NRS) and Athens Insomnia Scale (AIS). Postoperative pain scores were evaluated using the visual analog scale (VAS). Anxiety and depression were evaluated using the Hospital Anxiety and Depression Scale (HADS) on preoperative day 1 and on postoperative days (PODs) 1 and 3.

The primary outcome was the incidence of PSD, which was evaluated using the NRS and AIS on PODs 1 and 3. Postoperative sleep disturbance was defined as having an NRS score of 6 or higher or AIS score of 6 points or higher, indicating that sleep was repeatedly interrupted throughout the night, or even worse.^22,23^ The secondary outcomes included postoperative pain scores at rest and on movement at 24 hours and 48 hours (using VAS), postoperative hydromorphone consumption in the first 24 hours and total consumption within 48 hours, anxiety and depression scores on PODs 1 and 3 (using HADS), and the risk factors associated with PSD.

The NRS score ranges from 0 to 10, wherein 0 represents excellent or good sleep and 10 represents the inability to fall asleep all night. The AIS is a self-rated psychometric questionnaire to quantify sleep difficulties according to the *International Statistical Classification of Diseases and Related Health Problems, Tenth Revision* criteria.^24^ The AIS consists of 8 items: waking up at night, sleep induction, final awakening, total sleep duration, sleep quality, well-being, functional ability, and daytime sleepiness. The AIS score ranges from 0 to 24 points, and a total score of 6 points or higher indicates a diagnosis of insomnia.

The VAS score ranges from 0 (indicating no pain) to 10 (indicating intolerable pain) points. The HADS consists of 14 questions, with 7 items each for the anxiety and depression subscales. The score for each item ranges from 0 to 3 points, and scores are summed to yield a separate score for anxiety (HADS-A) and depression (HADS-D). Scores of 8 points or higher are diagnosed as depression or anxiety.

Postoperative hydromorphone consumption as well as postoperative complications, including nausea and vomiting, dizziness, itching, and nightmares, were noted and treated accordingly. In addition, surgery and anesthesia duration, consumption of intraoperative remifentanil, estimated infusion volume, blood loss and urine output, laryngeal mask removal time (the time from the end of surgery to airway removal of laryngeal mask), and length of stay in the postanesthesia care unit were also recorded.

### Statistical Analysis

The estimated sample size was calculated from the results of a preliminary study, wherein the incidence of PSD was 40% in gynecological laparoscopic surgery on POD 1. The expected effect size was calculated to detect a 50% incidence reduction in PSD after surgery, with a 2-sided α = .05 and 80% power; 164 patients were needed. Considering the possibility of loss to follow-up or consent withdrawals, 184 patients were included in this trial.

Statistical analysis was performed with SPSS, version 21.0 (IBM SPSS). The normal distribution of the variables was examined using the Kolmogorov-Smirnov test. Continuous data were presented as mean (SD) and compared using the unpaired, 2-tailed *t* test if distributed normally. Data that were not normally distributed were reported as median (IQR) and analyzed using the Mann-Whitney test. Categorical variables were reported as number (%) and compared using χ^2^ or Fisher exact test, as appropriate.

The results were analyzed using the per protocol principle because there was 1 incomplete piece of information. A logistic regression model was used to assess the potential risk factors associated with PSD. Two-sided *P* < .05 was considered to be statistically significant.

## Results

A total of 200 female patients were assessed for eligibility, and 16 patients were excluded based on the exclusion criteria. There was 1 patient with data loss in the control group on the third postoperative day (Figure 1). In total, 183 patients were randomized into 2 groups: control group (n = 91; median [IQR] age, 45 [35-49] years) and esketamine group (n = 92; median [IQR] age, 43 [32-49] years) (Table 1).

**Table 1.** Patient Demographic and Baseline Characteristics^a^

Characteristic	Patients, No. (%)	
	Control group (n = 91)	Esketamine group (n = 92)
Age, median (IQR), y	45 (35-49)	43 (32-49)
Height, median (IQR), cm	160 (158-163)	160 (158-163)
BMI, median (IQR)	23.8 (21.9-26.0)	23.2 (21.2-26.0)
ASA Physical Status Classification		
I	54 (59.3)	58 (63.0)
II	35 (38.5)	32 (34.8)
III	2 (2.2)	2 (2.2)
Surgery type		
Myomectomy	47 (51.6)	47 (51.1)
Hysterectomy	44 (48.4)	45 (48.9)
Diabetes	10 (10.9)	11 (12.0)
Hypertension	15 (16.5)	12 (13.0)
Anemia	28 (30.8)	32 (34.8)
Thyroid disease	4 (4.4)	2 (2.2)
Liver disease	4 (4.4)	2 (2.2)
Kidney disease	2 (2.2)	3 (3.3)
Drinking status	5 (5.5)	4 (4.3)
Smoking status	3 (3.3)	4 (4.3)
PSQI, median (IQR)	3 (3-4)	3 (3-4)
HADS-A score, median (IQR)	7 (5-8)	7 (5-8)
HADS-D score, median (IQR)	3 (2-4)	3 (2-4)
Sleep quality, median (IQR)		
AIS score	3 (2-6)	3 (2-6)
NRS score	5 (4-6)	5 (5-6)

Abbreviations: AIS, Athens Insomnia Scale; ASA, American Society of Anesthesiologists; BMI, body mass index (calculated as weight in kilograms divided by height in meters squared); HADS-A, Hospital Anxiety and Depression Scale-Anxiety; HADS-D, Hospital Anxiety and Depression Scale-Depression; NRS, numeric rating scale; PSQI, Pittsburgh Sleep Quality Index.

^a^ Data presented as median (IQR) were compared using the Mann-Whitney test. Data reported as the number of patients (%) were compared using the Pearson χ^2^ test or Fisher exact test.

The demographic data in terms of age, body mass index, ASA classification, surgery type (hysterectomy or myomectomy), preexisting medical conditions, smoking status, and drinking status were similar in both groups. Furthermore, there were no significant differences between the 2 groups for preoperative HADS scores for anxiety and depression, PSQI for sleep disorder, and NRS and AIS scores for sleep quality (Table 1).

Surgery or anesthesia duration, infusion volume, estimated blood loss, and urine output were not different between the 2 groups. The median (IQR) consumption of intraoperative remifentanil was significantly lower in the esketamine group than in the control group (0.14 [0.12-0.16] vs 0.15 [0.13-0.17] μg/kg/min; *P* = .003). The esketamine group had a longer median (IQR) laryngeal mask removal time (14 [8-20] vs 10 [8-14] minutes; *P* = .01) and time in the postanesthesia care unit (36 [32-40] vs 32 [30-34] minutes; *P* < .001) than the control group (eTable 1 in Supplement 2).

The median (IQR) NRS (5 [4-5] vs 5 [5-6]; *P* < .001) and AIS (2 [2-3] vs 3 [2-6] points; *P* = .01) subjective sleep quality scores in the esketamine group were significantly lower compared with the control group on POD 1. However, the NRS and AIS scores were not significantly different on POD 3 (Table 2).

**Table 2.** Sleep Quality, Anxiety, and Depression Scores on Postoperative Days (PODs) 1 and 3^a^

	Median (IQR)		*P* value
	Control group (n = 91)	Esketamine group (n = 92)	
**POD 1**			
Sleep quality			
AIS score	3 (2-6)	2 (2-3)	.01
NRS score	5 (5-6)	5 (4-5)	<.001
PSD, No. (%)	40 (44.0)	21 (22.8)	.002
HADS-A score	5 (4-6)	5 (4-6)	.71
HADS-D score	2 (2-3)	2 (2-3)	.43
**POD 3**			
Sleep quality			
AIS score	3 (2-3)	2 (2-3)	.06
NRS score	4 (4-5)	4 (4-5)	.05
PSD, No. (%)	18 (19.8)	7 (7.6)	.02
HADS-A score	4 (4-5)	4 (3-5)	.68
HADS-D score	2 (2-2)	2 (2-3)	.75

Abbreviations: AIS, Athens Insomnia Scale; HADS-A, Hospital Anxiety and Depression Scale-Anxiety; HADS-D, Hospital Anxiety and Depression Scale-Depression; NRS, numeric rating scale; PSD, postoperative sleep disturbance.

^a^ Data presented as median (IQR) were compared using the Mann-Whitney test. Data reported as the number of patients (%) were compared using the Pearson χ^2^ test or Fisher exact test.

**Figure 2.**
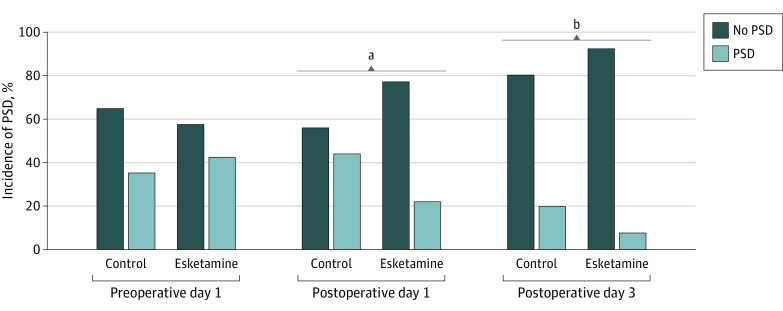
Incidence of Postoperative Sleep Disturbance (PSD) on Preoperative Day 1, Postoperative Day 1, and Postoperative Day 3 ^a^*P* < .01 esketamine vs control groups. ^b^*P* < .001 esketamine vs control groups.

The esketamine group had a lower incidence of PSD on POD 1 (22.8% vs 44.0%; odds ratio [OR], 0.38 [95% CI, 0.20-0.72]; *P* = .002) and POD 3 (7.6% vs 19.8%; OR, 0.33 [95% CI, 0.13-0.84]; *P* = .02) compared with the control group. Moreover, compared with the preoperative baseline value, the incidence of PSD in the control group did not significantly increase. In contrast, the incidence of PSD in the esketamine group on POD 1 decreased significantly compared with baseline (42.4% vs 22.8%; *P* = .007). There were no differences in HADS-A and HADS-D scores on POD 1 and POD 3 (Table 2 and Figure 2).

There were no significant differences in VAS scores for pain at rest at 24 hours and 48 hours after surgery. However, the median (IQR) VAS scores for movement were significantly lower in the esketamine group vs control group at 24 hours (3 [3-4] vs 4 [3-5] points; *P* < .001) and 48 hours (2 [2-3] vs 3 [2-3] points; *P* = .002) after surgery. The median (IQR) hydromorphone consumption in the first 24 hours after surgery (3.0 [2.8-3.3] mg vs 3.2 [2.9-3.4] mg; *P* = .04) and total consumption 48 hours after surgery (5.9 [5.5-6.5] mg vs 6.1 [5.7-6.7] mg; *P* = .13) were lower in the esketamine group than in the control group (eTable 2 in Supplement 2).

The incidence of postoperative vomiting and nausea, dizziness, itching, and nightmare was not significantly different between the 2 groups. Only 1 patient reported having a nightmare (eTable 3 in Supplement 2).

**Figure 3.**
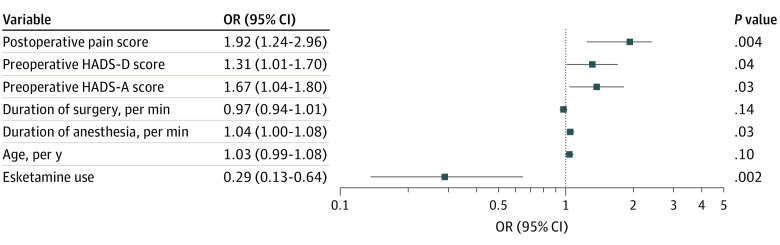
Forest Plot of Factors Analyzed for Association With Incidence of Postoperative Sleep Disturbance in Multivariable Logistic Regression HADS-A indicates Hospital Anxiety and Depression Scale-Anxiety; HADS-D, Hospital Anxiety and Depression Scale-Depression; OR, odds ratio.

Multivariable logistic regression was used to explore the risk factors associated with PSD, and these were found to include preoperative depression and anxiety scores (HADS-D: OR, 1.31 [95% CI, 1.01-1.70]; HADS-A: OR, 1.67 [95% CI, 1.04-1.80]), duration of anesthesia (OR, 1.04; 95% CI, 1.00-1.08), and postoperative pain scores (OR, 1.92; 95% CI, 1.24-2.96) (Figure 3).

## Discussion

The main findings of this trial were as follows. First, postoperative sleep quality and PSD in the esketamine group were significantly lower compared with the control group, suggesting that esketamine can prevent poor postoperative sleep quality and PSD in patients who underwent gynecological laparoscopic surgery. Second, esketamine infusion did not alter the depression and anxiety scores from before the surgery because the scores were low after surgery. There were no significant differences in VAS scores for pain at rest. In contrast, the esketamine group had significantly lower VAS scores for pain on movement. Moreover, esketamine significantly attenuated hydromorphone consumption in the first 24 hours after surgery. Third, multivariable logistic regression showed that preoperative depression and anxiety scores, duration of anesthesia, and postoperative pain scores were all risk factors for PSD. Esketamine had a prophylactic effect against poor postoperative sleep quality and PSD in patients after surgery.

Postoperative sleep disturbance occurs frequently in older patients undergoing major surgery, whereas its incidence in patients undergoing gynecological surgery has increased to 31.4%,^23^ which is similar to findings in the present trial (44.0% in the control group on POD 1). Specifically, we found that esketamine significantly decreased the PSD incidence from 44.0% to 22.8% on POD 1 and from 19.8% to 7.6% on POD 3. A recent study reported that a subanesthetic dose (0.5-0.75 mg/kg) of (*R,S*)-ketamine produced an antidepressant effect and better sleep among patients in the intensive care unit.^25^ Collectively, these data suggest that perioperative esketamine infusion has a prophylactic effect on PSD after surgery.

Pain is a leading cause of PSD in patients after surgery.^8^ In this trial, the VAS score on movement in the esketamine group was lower compared with the control group. The esketamine group had significantly reduced opioid consumption compared with the control group. Given the analgesic properties of esketamine, the effect of esketamine on postoperative sleep quality and PSD may be associated with the reduction of opioid consumption in patients who underwent gynecological laparoscopic surgery. However, the association between perioperative opioid consumption and PSD development is currently contradictory.^26,27,28^

Multivariable logistic analysis showed that preoperative depression and anxiety scores, duration of anesthesia, and postoperative pain scores were associated with the occurrence of PSD, a finding that was in agreement with the results of previous studies.^29,30^ Poor sleep quality is often seen in older adults, whereas depression and anxiety are both psychiatric risk factors for PSD development.^8^ Thus, it is possible that anesthesia duration is a risk factor for PSD and that anesthetics, such as isoflurane, sevoflurane, and propofol, have a hypnosis function but are associated with PSD development.

The underlying mechanisms of the prophylactic effect of esketamine on postoperative sleep quality and PSD are currently unclear. After surgery, patients often experience an increase in proinflammatory cytokines, endothelial dysfunction, glycocalyx damage, and activation of neutrophils,^31,32^ resulting in PSD. Thus, the anti-inflammatory effect of esketamine may contribute to its prophylactic effect on PSD. Accumulating evidence suggests that (*R,S*)-ketamine can modulate circadian rhythms by regulating clock genes.^21,33,34^ Therefore, it is likely that the beneficial effect of esketamine on PSD is related to the modulation of the circadian rhythm system. Nonetheless, further studies are needed to confirm the role of circadian rhythms in the prophylactic effect of esketamine on PSD.

Bonaventura et al^35^ reported that, in addition to NMDAR (Ki = 0.8 μM), esketamine has a moderate affinity (Ki = 7 μM) with the μ-opioid receptor. Thus, it is reasonable that the esketamine group had lower consumption of intraoperative remifentanil, a short-acting μ-opioid agonist. Furthermore, it is likely that both NMDAR and μ-opioid receptor play a role in the prophylactic effect of esketamine on PSD after surgery.^36^ Further studies on opioid receptor antagonists are needed to ascertain the role of the opioid receptor in the prophylactic effect of esketamine on PSD.

### Limitations

This study has some limitations. First, there was a change in methods after trial commencement; one of the inclusion criteria changed from ASA I to II to ASA I to III compared with that in the registration protocol in the Chinese Clinical Trial Registry, but only 4 patients who were evaluated to have ASA III were included to reduce study time and were divided into 2 groups equally (2 in the esketamine group, 2 in the control group). Second, this trial was conducted in a single center, and thus multicenter studies are needed. Third, biological samples, such as blood and feces, were not collected; these samples could have helped determine the possible mechanism behind the beneficial effect of esketamine on PSD. Furthermore, there was no bispectral index monitoring during the operation in this trial because the association between ketamine-induced anesthesia and bispectral index was not high.^37^ Fourth, polysomnography and electroencephalography were not used to analyze the sleep quality among patients due to the complexity of monitoring, particularly in a trial with a large sample size.

## Conclusions

The results of this placebo-controlled randomized clinical trial revealed that intraoperative esketamine infusion improved the incidence of PSD in patients who underwent gynecological laparoscopic surgery. Further studies with a larger sample size are needed to confirm the prophylactic effect of esketamine on PSD.

## Notes

Supplement 1.
Trial Protocol

Supplement 2. **eTable 1.** Perioperative Data **eTable 2.** Pain Scores and Opioid Consumption at 24 h and 48 h Postoperatively **eTable 3.** Postoperative Complications on POD 1 and 3

Supplement 3.
Data Sharing Statement

## References

[1] Gögenur I, Wildschiøtz G, Rosenberg J. Circadian distribution of sleep phases after major abdominal surgery. Br J Anaesth. 2008;100(1):45-49. doi:10.1093/bja/aem340 18037670

[2] Chouchou F, Khoury S, Chauny JM, Denis R, Lavigne GJ. Postoperative sleep disruptions: a potential catalyst of acute pain? Sleep Med Rev. 2014;18(3):273-282. doi:10.1016/j.smrv.2013.07.002 24074687

[3] Su X, Wang DX. Improve postoperative sleep: what can we do? Curr Opin Anaesthesiol. 2018;31(1):83-88. doi:10.1097/ACO.0000000000000538 29120927 PMC5768217

[4] Lu Y, Li YW, Wang L, . Promoting sleep and circadian health may prevent postoperative delirium: a systematic review and meta-analysis of randomized clinical trials. Sleep Med Rev. 2019;48:101207. doi:10.1016/j.smrv.2019.08.001 31505369

[5] Wang X, Hua D, Tang X, . The role of perioperative sleep disturbance in postoperative neurocognitive disorders. Nat Sci Sleep. 2021;13:1395-1410. doi:10.2147/NSS.S320745 34393534 PMC8354730

[6] O’Gara BP, Gao L, Marcantonio ER, Subramaniam B. Sleep, pain, and cognition: modifiable targets for optimal perioperative brain health. Anesthesiology. 2021;135(6):1132-1152. doi:10.1097/ALN.0000000000004046 34731233 PMC8578455

[7] Ibala R, Mekonnen J, Gitlin J, . A polysomnography study examining the association between sleep and postoperative delirium in older hospitalized cardiac surgical patients. J Sleep Res. 2021;30(5):e13322. doi:10.1111/jsr.13322 33759264 PMC8637551

[8] Luo M, Song B, Zhu J. Sleep disturbances after general anesthesia: current perspectives. Front Neurol. 2020;11:629. doi:10.3389/fneur.2020.00629 32733363 PMC7360680

[9] Hashimoto K. Molecular mechanisms of the rapid-acting and long-lasting antidepressant actions of (R)-ketamine. Biochem Pharmacol. 2020;177:113935. doi:10.1016/j.bcp.2020.113935 32224141

[10] Wei Y, Chang L, Hashimoto K. Molecular mechanisms underlying the antidepressant actions of arketamine: beyond the NMDA receptor. Mol Psychiatry. 2022;27(1):559-573. doi:10.1038/s41380-021-01121-1 33963284 PMC8960399

[11] Zarate CA Jr, Singh JB, Carlson PJ, . A randomized trial of an N-methyl-D-aspartate antagonist in treatment-resistant major depression. Arch Gen Psychiatry. 2006;63(8):856-864. doi:10.1001/archpsyc.63.8.856 16894061

[12] Murrough JW, Iosifescu DV, Chang LC, . Antidepressant efficacy of ketamine in treatment-resistant major depression: a two-site randomized controlled trial. Am J Psychiatry. 2013;170(10):1134-1142. doi:10.1176/appi.ajp.2013.13030392 23982301 PMC3992936

[13] Singh JB, Fedgchin M, Daly E, . Intravenous esketamine in adult treatment-resistant depression: a double-blind, double-randomization, placebo-controlled study. Biol Psychiatry. 2016;80(6):424-431. doi:10.1016/j.biopsych.2015.10.018 26707087

[14] Wichniak A, Wierzbicka A, Jernajczyk W. Sleep as a biomarker for depression. Int Rev Psychiatry. 2013;25(5):632-645. doi:10.3109/09540261.2013.812067 24151807

[15] Asarnow LD. Depression and sleep: what has the treatment research revealed and could the HPA axis be a potential mechanism? Curr Opin Psychol. 2020;34:112-116. doi:10.1016/j.copsyc.2019.12.002 31962280 PMC8412030

[16] Liverant GI, Arditte Hall KA, Wieman ST, Pineles SL, Pizzagalli DA. Associations between insomnia and reward learning in clinical depression. Psychol Med. 2021;1-10. doi:10.1017/S003329172100026X 33634765

[17] Lee MJ, Nho WY, Jung H, . High prevalence of depression and sleep-wake disorders among female emergency medicine residents in South Korea. Ann Med. 2022;54(1):846-855. doi:10.1080/07853890.2022.2053568 35348012 PMC8967212

[18] Wang M, Zhang B, Zhou Y, . Sleep improvement is associated with the antidepressant efficacy of repeated-dose ketamine and serum BDNF levels: a post-hoc analysis. Pharmacol Rep. 2021;73(2):594-603. doi:10.1007/s43440-020-00203-1 33387333

[19] Song B, Zhu JC. Mechanisms of the rapid effects of ketamine on depression and sleep disturbances: a narrative review. Front Pharmacol. 2021;12:782457. doi:10.3389/fphar.2021.782457 34970147 PMC8712478

[20] Song B, Zhu J. A novel application of ketamine for improving perioperative sleep disturbances. Nat Sci Sleep. 2021;13:2251-2266. doi:10.2147/NSS.S341161 34992482 PMC8715868

[21] Kohtala S, Alitalo O, Rosenholm M, Rozov S, Rantamäki T. Time is of the essence: coupling sleep-wake and circadian neurobiology to the antidepressant effects of ketamine. Pharmacol Ther. 2021;221:107741. doi:10.1016/j.pharmthera.2020.107741 33189715

[22] Cai J, Chen Y, Hao X, . Effect of intraoperative dexmedetomidine dose on postoperative first night sleep quality in elderly surgery patients: a retrospective study with propensity score-matched analysis. Front Med (Lausanne). 2020;7:528. doi:10.3389/fmed.2020.00528 33117823 PMC7574233

[23] Duan G, Wang K, Peng T, Wu Z, Li H. The effects of intraoperative dexmedetomidine use and its different dose on postoperative sleep disturbance in patients who have undergone non-cardiac major surgery: a real-world cohort study. Nat Sci Sleep. 2020;12:209-219. doi:10.2147/NSS.S239706 32210652 PMC7075348

[24] Song B, Li Y, Teng X, Li X, Yang Y, Zhu J. Comparison of morning and evening operation under general anesthesia on intraoperative anesthetic requirement, postoperative sleep quality, and pain: a randomized controlled trial. Nat Sci Sleep. 2020;12:467-475. doi:10.2147/NSS.S257896 32765143 PMC7371604

[25] Giri AR, Kaur N, Yarrarapu SNS, . Novel management of depression using ketamine in the intensive care unit. J Intensive Care Med. 2022;8850666221088220. doi:10.1177/08850666221088220 35313768

[26] Cronin AJ, Keifer JC, Davies MF, King TS, Bixler EO. Postoperative sleep disturbance: influences of opioids and pain in humans. Sleep. 2001;24(1):39-44. doi:10.1093/sleep/24.1.39 11204052

[27] Eacret D, Veasey SC, Blendy JA. Bidirectional relationship between opioids and disrupted sleep: putative mechanisms. Mol Pharmacol. 2020;98(4):445-453. doi:10.1124/mol.119.119107 32198209 PMC7562980

[28] Ellis JD, Mayo JL, Gamaldo CE, Finan PH, Huhn AS. Worsening sleep quality across the lifespan and persistent sleep disturbances in persons with opioid use disorder. J Clin Sleep Med. 2022;18(2):587-595. doi:10.5664/jcsm.9676 34569924 PMC8805005

[29] Paulose JK, Wang C, O’Hara BF, Cassone VM. The effects of aging on sleep parameters in a healthy, melatonin-competent mouse model. Nat Sci Sleep. 2019;11:113-121. doi:10.2147/NSS.S214423 31496853 PMC6697669

[30] Haack M, Simpson N, Sethna N, Kaur S, Mullington J. Sleep deficiency and chronic pain: potential underlying mechanisms and clinical implications. Neuropsychopharmacology. 2020;45(1):205-216. doi:10.1038/s41386-019-0439-z 31207606 PMC6879497

[31] Alam A, Hana Z, Jin Z, Suen KC, Ma D. Surgery, neuroinflammation and cognitive impairment. EBioMedicine. 2018;37:547-556. doi:10.1016/j.ebiom.2018.10.021 30348620 PMC6284418

[32] Margraf A, Ludwig N, Zarbock A, Rossaint J. Systemic inflammatory response syndrome after surgery: mechanisms and protection. Anesth Analg. 2020;131(6):1693-1707. doi:10.1213/ANE.0000000000005175 33186158

[33] Orozco-Solis R, Montellier E, Aguilar-Arnal L, . A circadian genomic signature common to ketamine and sleep deprivation in the anterior cingulate cortex. Biol Psychiatry. 2017;82(5):351-360. doi:10.1016/j.biopsych.2017.02.1176 28395871 PMC5660920

[34] Sato S, Bunney B, Mendoza-Viveros L, . Rapid-acting antidepressants and the circadian clock. Neuropsychopharmacology. 2022;47(4):805-816. doi:10.1038/s41386-021-01241-w 34837078 PMC8626287

[35] Bonaventura J, Lam S, Carlton M, . Pharmacological and behavioral divergence of ketamine enantiomers: implications for abuse liability. Mol Psychiatry. 2021;26(11):6704-6722. doi:10.1038/s41380-021-01093-2 33859356 PMC8517038

[36] Schatzberg AF. Mechanisms of action of ketamine and esketamine. Am J Psychiatry. 2021;178(12):1130. doi:10.1176/appi.ajp.2021.21060653 34855448

[37] Hans P, Dewandre PY, Brichant JF, Bonhomme V. Comparative effects of ketamine on Bispectral Index and spectral entropy of the electroencephalogram under sevoflurane anaesthesia. Br J Anaesth. 2005;94(3):336-340. doi:10.1093/bja/aei047 15591328

