# Supplementary material for: Effect of Intraoperative Esketamine Infusion on Postoperative Sleep Disturbance After Gynecological Laparoscopy: A Randomized Clinical Trial
Source: JAMA Netw Open. 2022 Dec 1;5(12):e2244514. doi: 10.1001/jamanetworkopen.2022.44514 (PMC9716381; doi:10.1001/jamanetworkopen.2022.44514)
Supplement: Supplement 1. — Trial Protocol [file jamanetwopen-e2244514-s001.pdf]

Effect of operative esketamine infusion on postoperative sleep disturbance among patients undergoing gynecological laparoscopic surgery: a randomized clinical trial.

Study Protocol

Clinical site:

The First Affiliated Hospital of Zhengzhou University, Henan, China

Data management and statistical site:

Department of Anesthesiology, Pain and Perioperative Medicine, The First Affiliated Hospital of Zhengzhou University, Henan, China

Data:

Original protocol date: August, 2021

Final protocol date: April, 2022

Contents

1 Committee composition 1
1.1 Protocol committee 1
1.2 Steering committee 1
1.3 Data coordination committee 1
1.4 Publication committee 2
2 Study contacts and organizations 2
2.1 Study contacts 2
2.2 Recruiting site 2
3 Background 2
4 Study hypotheses 3
5 Methodology 3
5.1 Study design 3
5.2 Randomization and blinding 3
5.3 Sample size 3
5.4 Participant recruitment, screening and group assignment 4
5.5 Inclusion criteria 4
5.8 Exclusion criteria 4
6 Outcome measurement 5
6.1 Primary outcome 5
6.2 Secondary outcomes 5
7 Interventions 6
8 Anesthesia Technique 6
9 Quality control 6
10 Statistical analysis 6
References 7

1 Committee composition

1.1 Protocol committee

Table 1 Protocol committee

Name	Affiliation	Email address
Di Qiu	The First Affiliated Hospital of Zhengzhou University	qd15136760769@163.com
Xing-Ming Wang	The First Affiliated Hospital of Zhengzhou University and Division of Clinical Neuroscience, Chiba University Center for Forensic Mental Health	xingmingwang@chiba-u.jp
Jin-Jin Yang	The First Affiliated Hospital of Zhengzhou University	18845145049@163.com
Sai Chen	The First Affiliated Hospital of Zhengzhou University	13781126313@163.com
Cai-Bao Yue	The First Affiliated Hospital of Zhengzhou University	Yuecb1987@163.com
Kenji Hashimoto	Division of Clinical Neuroscience, Chiba University Center for Forensic Mental Health	hashimoto@faculty.chiba-u.jp
Jian-Jun Yang	The First Affiliated Hospital of Zhengzhou University	yjyangjj@163.com

1.2 Steering committee

Table 2 Steering committee

Name	Affiliation	Email address
Di Qiu	The First Affiliated Hospital of Zhengzhou University	qd15136760769@163.com
Xing-Ming Wang	The First Affiliated Hospital of Zhengzhou University and Division of Clinical Neuroscience, Chiba University Center for Forensic Mental Health	xingmingwang@chiba-u.jp
Kenji Hashimoto	Division of Clinical Neuroscience, Chiba University Center for Forensic Mental Health	hashimoto@faculty.chiba-u.jp
Jian-Jun Yang	The First Affiliated Hospital of Zhengzhou University	yjyangjj@163.com

1.3 Data coordination committee

Table 3 Data coordination committee

Name	Affiliation	Email address
Di Qiu	The First Affiliated Hospital of Zhengzhou University	qd15136760769@163.com
Xing-Ming Wang	The First Affiliated Hospital of Zhengzhou University and Division of Clinical Neuroscience, Chiba University Center for Forensic Mental Health	xingmingwang@chiba-u.jp
Jin-Jin Yang	The First Affiliated Hospital of Zhengzhou University	18845145049@163.com
Cai Chen	The First Affiliated Hospital of Zhengzhou University	13781126313@163.com
Cai-Bao Yue	The First Affiliated Hospital of Zhengzhou University	Yuecb1987@163.com

1.4 Publication committee

Members of the publication committee include Di Qiu, Xing-Ming Wang, Jin-Jin Yang, Sai Chen, Cai-Bao Yue, Kenji Hashimoto, Jian-Jun Yang.

2 Study contacts and organizations

2.1 Study contacts

Principal investigator for trial
Jian-Jun Yang, MD PhD
Department of Anesthesiology, Pain and Perioperative Medicine, The First Affiliated Hospital of Zhengzhou University, No. 1 East Jianshe Road, Zhengzhou, 450052, China
Email: yjyangjj@163.com

2.2 Recruiting site

The First Affiliated Hospital of Zhengzhou University

3 Background

Postoperative sleep disturbance (PSD), including sleep deprivation, circadian disruption, and abnormal architecture, frequently occurs in patients after surgery (1-3). PSD could lead to postoperative delirium and cognitive impairment, exacerbate acute postoperative pain, and delay postoperative recovery (2, 4-7). Pharmacological intervention such as short-acting non-benzodiazepine zolpidem and melatonin has been often used to treat postoperative sleep quality (8). However，there are no studies reporting prophylactic effects for PSD.

(*R*,*S*)-ketamine, an *N*-methyl-D-aspartate receptor (NMDAR) antagonist, is a racemic mixture of equal amounts of (*R*)-ketamine (or arketamine) and (*S*)-ketamine (or esketamine), with esketamine having greater affinity for the NMDA receptor (9,10). Subanesthetic dose of (*R*,*S*)-ketamine (0.5 mg/kg, 40-min intravenous infusion) is well known to produce rapid-acting and sustained antidepressant actions in treatment-resistant patients with major depressive disorder (MDD) (11,12). Esketamine for anesthesia has been used some countries including China. Subanesthetic dose (0.2 and 0.4 mg/kg, intravenous infusion) of esketamine has been demonstrated rapid and robust antidepressant efficacy in treatment resistant patients with MDD although transient psychotomimetic and dissociative side effects were shown after infusion (13).

PSD is often shown with depression in postoperative patients (14-17). In addition to antidepressant actions, infusion of (*R*,*S*)-ketamine could improve the sleep disturbance in MDD patients with a sleep problem, suggesting potential of (*R*,*S*)-ketamine for sleep disturbance (18-20). It is suggested that antidepressant effects of (*R*,*S*)-ketamine are connected with the neurobiology of wake, sleep, and circadian rhythms (21). However, it is unknown whether intravenous infusion of low dose of esketamine intraoperatively can reduce the incidence of PSD in patient with surgery.

4 Study hypotheses

The following hypotheses will be tested:

H1: There is a significant difference in the incidence of PSD on POD 1 and POD 3 in patient with surgery between the ketamine group and the control group.

H0: There is no difference in the incidence of PSD on POD 1 and POD 3 in patient with surgery between the ketamine group and the control group.

5 Methodology

5.1 Study design

This is a single-center, double-blind, randomized controlled trial in the First Affiliated Hospital of Zhengzhou University in China. Participants who underwent gynecological laparoscopic surgery will be randomized into esketamine group or control group (saline) in a 1:1 ratio.

5.2 Randomization and blinding

The blocked randomization sequence will be computer-generated by an independent researcher (Cai-Bao Yue, the First Affiliated Hospital of Zhengzhou University in China), who is not involved in the implementation and statistical analysis of the trial. For allocation concealment, assignments were concealed in sealed envelopes, which were sequentially handed to a nurse who was not involved in the study. When eligible patients are enrolled into the trial, envelopes will be successively opened by the nurse. Both esketamine and saline were prepared in identical 20-mL syringes by the same nurse. Anesthesiologists involved in the patient management and had participated in the postoperative follow-up were blinded to the group assignment. Esketamine (50 mg) was diluted to a total of 20 mL with saline solution. Group assignments were not revealed until patients were discharged from the hospital.

5.3 Sample size

In this study, the estimated sample size was calculated based on the results of our preliminary study, wherein the incidence of PSD was 40% in gynecological laparoscopic surgery. The expected effect size was calculated to detect a 50% incidence reduction in PSD after surgery with a two-sided $\alpha = 0.05$ with 80% power; 164 patients were needed. Considering the possibility of loss to follow-up or consent withdrawals, the recruitment goal was set at 184 patients.

5.4 Participant recruitment, screening and group assignment

Participants (ASA I-III) who underwent gynecological laparoscopic surgery will be recruited at the First Affiliated Hospital of Zhengzhou University in China. Written informed consent will be provided by each patient before the enrollment. Enrolled patients were randomly assigned to esketamine group or control group in a 1:1 ratio. The evaluators will record the data on the electronic case report through the whole trial period. The participant flow was shown in Figure 1 and Figure 2. The ethics boards of The First Affiliated Hospital of Zhengzhou University have approved this study.

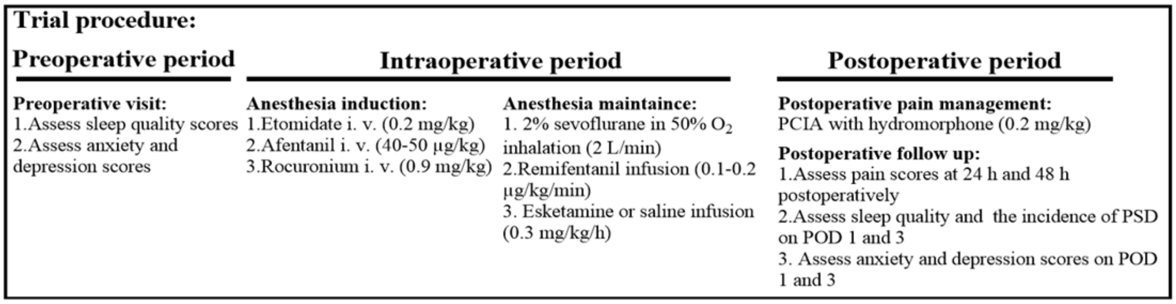

Figure 1. Procedure of management and visit

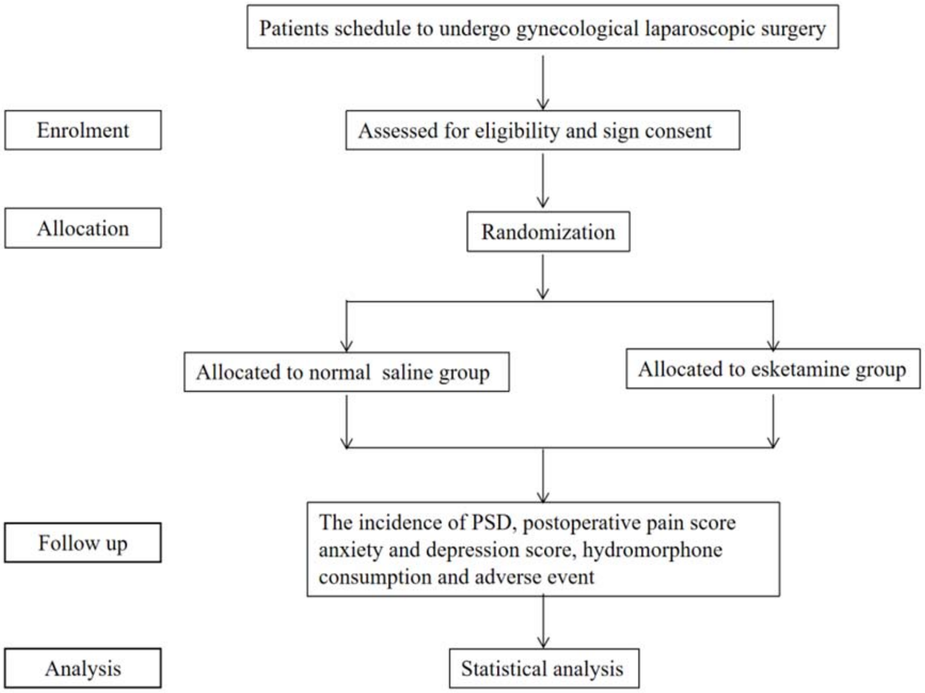

Figure 2 | The Flow diagram

5.5 Inclusion criteria

Participants will be included in the study if they meet the following criteria.

1) Age 18 to 65 years.
2) American Society of Anesthesiologists Physical Status I-III.
3) BMI 18-30 kg/m^2.
4) Schedule to undergo gynecological laparoscopic surgery.

5.6 Exclusion criteria

Participants will be excluded from the study if they met the following criteria.

1) Patient refusal to participate in the study.
2) Body mass index (BMI) > 30 kg/m^{2}.
3) Preoperative Pittsburgh Sleep Quality Index (PSQI) > 7.
4) Recent history of drug abuse.
5) Contraindications or allergic to esketamine.
6) Cognitive dysfunction or unable to communicate.
7) Inability to use patient-controlled intravenous analgesia (PCIA).

6 Outcome measurement

6.1 Primary outcome

The primary outcome was the incidence of PSD on postoperative days 1 and 3. The PSD is defined as Numerical Rating Scale (NRS) ≥ 6 or Athens Insomnia Scale (AIS) ≥ 6, indicating that sleep is repeatedly interrupted throughout the night, or even worse. The AIS consists of 8 items—waking up at night, sleep induction, final awakening, total sleep duration, sleep quality, well-being, functional ability, and daytime sleepiness.

6.2 Secondary outcomes

1.Postoperative pain score at 24h and 48h after surgery, assessed using the visual analogue scale (VAS), this is a scale ranging from one to ten, in which one indicates 'no pain' and ten indicates 'Unbearable pain'.
2.Anxiety and depression score on 1 day before surgery, postoperative day 1 and 3, measured using the Hospital Anxiety and Depression Scale (HADS), which composed of 14 questions with 7 items each for anxiety and depression. Scores ≥ 8 are considered positive for both depression and anxiety scales.
3.Postoperative hydromorphone consumption in the first 24 h and total consumption within 48 h as well as postoperative complications including postoperative nausea and vomiting, dizziness, itching, and nightmare.
4. Other outcomes including demographic information such as age, height, BMI, ASA classification, smoking, drinking alcohol and comorbidities; and intraoperative information such as surgery and anesthesia duration, laryngeal mask removal time, infusion volume, estimated blood loss and urine output.

7 Interventions

Patients in both esketamine group and control group will receive similar intraoperative monitoring and anesthesia management. Patients in esketamine group will receive a continuous infusion of esketamine (0.3 mg/kg/h) intraoperatively, and patients in control group will receive an equivalent volume of saline.

8 Anesthesia Technique

Routine intraoperative monitoring was established including pulse oximetry, electrocardiography and noninvasive blood pressure. Anesthesia was induced with etomidate (0.2 mg/kg), alfentanil (40-50

μg/kg), followed by rocuronium (0.9 mg/kg) to facilitate the insertion of laryngeal mask airway (Ambu AuraGain, Baltorpbakken, Denmark). Anesthesia was maintained with remifentanil (0.1-0.2 μg/kg/min) and 2% sevoflurane in 50% O2 (2 L/min), and the remifentanil infusion rate was titrated to keep heart rate and arterial blood pressure within 20% of baseline. Maintain end-tidal carbon dioxide partial pressure between 35-45mmHg. Palonosetron (0.25 mg) was injected intravenously at the end of surgery for prevention of postoperative nausea and vomiting. After completing surgery, the laryngeal mask was pulled out when adequate muscle strength was established. Postoperative pain was managed using hydromorphone (0.2 mg/kg in 200 ml saline) by PCIA pump: background infusion 2 ml/h for 48 h, bolus 2 ml, lockout time 10 min. Flurbiprofen axetil (50 mg) was intravenously administered as a rescue analgesic to patients if the pain scores＞4.

9 Quality control

Both paper files and electronic documents will be preserved for at least 5 years after publication. If readers have any questions, they can contact the corresponding author for access to the original data. Patient information will remain anonymous, including name, ID number and telephone number. The protocol will be reviewed and revised by experts in anesthesia management and statistics. We will perform a pre-specified standard operating procedure, which includes screening patients, improve relevant management, and standardize the follow-up visit, assessing outcomes and data management. The ethics committee of The First Affiliated Hospital of Zhengzhou University will audit trial conduct per 12 months.

10 Statistical analysis

Patients' baseline characteristics will be summarized based on groups. Continuous variables will be described using the mean (standard deviation), or the median (interquartile range) if the normality assumption is violated. Student's t text or Mann–Whitney U test (if normality is violated) will be used for comparison of continuous variables among the two groups. Categorical variables will be described using the frequency (percentage) and compared using the chi-squared test or or Fisher exact test, as appropriate.

For the primary comparison, the chi-squared test will be used for the incidence of postoperative sleep disturbance. For the secondary outcomes, Student's t test, Chi-squared test, Fisher's exact test or the Mann–Whitney U test will be used to test the difference of the outcomes including postoperative pain score, anxiety and depression score, postoperative hydromorphone consumption and adverse events, between groups according to the distribution of variables. There is no interim analysis or additional analysis in this trial.

All efficacy analyses will be performed using the per-protocol, which includes all randomized patients except for one missing data. All analyses will be performed using SPSS version 21.0 (IBM SPSS Statistics, USA).

The level of significance will be established at $\alpha < 0.05$ with a two-sided test.

References

1. Gögenur I, Wildschiøtz G, Rosenberg J: Circadian distribution of sleep phases after major

abdominal surgery. *Br J Anaesth* 2008; 100(1):45-49.
2. Chouchou F, Khoury S, Chauny J M, et al: Postoperative sleep disruptions: a potential catalyst of acute pain? *Sleep Med Rev* 2014; 18(3):273-282.
3. Su X, Wang DX: Improve postoperative sleep: what can we do? *Curr Opin Anaesthesiol* 2018; 31(1):83-88.
4. Lu Y, Li YW, Wang L, et al: Promoting sleep and circadian health may prevent postoperative delirium: A systematic review and meta-analysis of randomized clinical trials. *Sleep Med Rev* 2019; 48:101207.
5. Wang X, Hua D, Tang X, et al: The role of perioperative sleep disturbance in postoperative neurocognitive disorders. *Nat Sci Sleep* 2021; 13:1395-1410.
6. O'gara BP, Gao L, Marcantonio ER, et al: Sleep, pain, and cognition: modifiable targets for optimal perioperative brain health. *Anesthesiology* 2021; 135(6):1132-1152.
7. Ibala R, Mekonnen J, Gitlin J, et al: A polysomnography study examining the association between sleep and postoperative delirium in older hospitalized cardiac surgical patients. *J Sleep Res* 2021; 30(5):e13322.
8. Luo M, Song B, Zhu J: Sleep disturbances after general anesthesia: current perspectives. *Front Neurol* 2020; 11:629.
9. Hashimoto K: Molecular mechanisms of the rapid-acting and long-lasting antidepressant actions of (*R*)-ketamine. *Biochem Pharmacol* 2020; 177:113935.
10. Wei Y, Chang L, Hashimoto K: Molecular mechanisms underlying the antidepressant actions of arketamine: beyond the NMDA receptor. *Mol Psychiatry* 2022; 27(1):559-573.
11. Zarate CA Jr, Singh JB, Carlson PJ, et al: A randomized trial of an N-methyl-D-aspartate antagonist in treatment-resistant major depression. *Arch Gen Psychiatry* 2006; 63(8):856-864.
12. Murrough JW, Iosifescu DV, Chang LC, et al: Antidepressant efficacy of ketamine in treatment-resistant major depression: a two-site randomized controlled trial. *Am J Psychiatry* 2013; 170(10):1134-42.
13. Singh JB, Fedgchin M, Daly E, et al: Intravenous esketamine in adult treatment-resistant depression: A double-blind, double-randomization, placebo-controlled study. *Biol Psychiatry* 2016; 80(6):424-431.
14. Wichniak A, Wierzbicka A, Jernajczyk W: Sleep as a biomarker for depression. *Int Rev Psychiatry* 2013; 25(5):632-645.
15. Asarnow LD: Depression and sleep: what has the treatment research revealed and could the HPA axis be a potential mechanism? *Curr Opin Psychol* 2020; 34:112-116.
16. Liverant GI, Arditte Hall KA, Wieman ST, et al: Associations between insomnia and reward learning in clinical depression. *Psychol Med* 2021 Feb 26;1-10. doi: 10.1017/S003329172100026X.
17. Lee MJ, Nho WY, Jung H, et al: High prevalence of depression and sleep-wake disorders among female emergency medicine residents in South Korea. *Ann Med* 2022; 54(1):846-855.
18. Wang M, Zhang B, Zhou Y, et al: Sleep improvement is associated with the antidepressant efficacy of repeated-dose ketamine and serum BDNF levels: a post-hoc analysis. *Pharmacol Rep* 2021; 73(2):594-603.

19. Song B, Zhu JC: Mechanisms of the rapid effects of ketamine on depression and sleep disturbances: A narrative review. *Front Pharmacol* 2021; 12:782457.
20. Song B, Zhu J: A novel application of ketamine for improving perioperative sleep disturbances. *Nat Sci Sleep* 2021; 13:2251-2266.
21. Kohtala S, Alitalo O, Rosenholm M, et al: Time is of the essence: Coupling sleep-wake and circadian neurobiology to the antidepressant effects of ketamine. *Pharmacol Ther* 2021; 221:107741.
