# Supplementary material for: Effect of Intraoperative Esketamine Infusion on Postoperative Sleep Disturbance After Gynecological Laparoscopy: A Randomized Clinical Trial
Source: JAMA Netw Open. 2022 Dec 1;5(12):e2244514. doi: 10.1001/jamanetworkopen.2022.44514 (PMC9716381; doi:10.1001/jamanetworkopen.2022.44514)
Supplement: Supplement 2. — eTable 1. Perioperative Data eTable 2. Pain Scores and Opioid Consumption at 24 h and 48 h Postoperatively eTable 3. Postoperative Complications on POD 1 and 3 [file jamanetwopen-e2244514-s002.pdf]

Supplemental Online Content

Qiu D, Wang XM, Yang JJ, et al. Effect of intraoperative esketamine infusion on postoperative sleep disturbance after gynecological laparoscopy: a randomized clinical trial. *JAMA Netw Open*. 2022;5(12):e2244514. doi:10.1001/jamanetworkopen.2022.44514

eTable 1. Perioperative Data

eTable 2. Pain Scores and Opioid Consumption at 24 h and 48 h Postoperatively

eTable 3. Postoperative Complications on POD 1 and 3

This supplemental material has been provided by the authors to give readers additional information about their work.

© 2022 Qiu D et al. *JAMA Network Open.*

eTable 1. Perioperative Data

	Control Group, median (IQR, n=91)	Esketamine Group, median (IQR, n=92)	*P*
Duration of anesthesia (min)	110 (90, 143)	121 (100, 142)	0.09
Duration of surgery (min)	85 (67, 114)	95 (75.5, 118.5)	0.06
Remifentanil dosage (μg/kg/min)	0.15 (0.13, 0.17)	0.14 (0.12, 0.16)	0.003
Infusion volume (mL)	1300 (1000, 1500)	1300 (1025, 1500)	0.68
Estimated blood loss (mL)	30 (20, 30)	30 (20, 30)	0.12
Urine volume (mL)	150 (80, 200)	150 (100, 287.5)	0.21
Laryngeal mask removal time (min)	10 (8, 14)	14 (8, 20)	0.01
Time in PACU (min)	32 (30, 34)	36 (32, 40)	< 0.001

Data presented as median (interquartile range, IQR) were compared using the Mann–Whitney U test or Fisher exact test.

Abbreviations: PACU, postanesthesia care unit.

© 2022 Qiu D et al. *JAMA Network Open.*

eTable 2. Pain Scores and Opioid Consumption at 24 h and 48 h Postoperatively

	Control group, median (IQR, n=91)	Esketamine group median (IQR, n=92)	*P*
24 h postoperatively			
VAS (R)	3 (2, 3)	2 (1, 3)	0.09
VAS (M)	4 (3, 5)	3 (3, 4)	$<$0.001
Hydromorphone consumption (mg)	3.2 (2.9, 3.4)	3.0 (2.8, 3.3)	0.04
48 h postoperatively			
VAS (R)	1 (1, 2)	1 (1, 2)	0.14
VAS (M)	3 (2, 3)	2 (2, 3)	0.002
Hydromorphone consumption (mg)	6.1 (5.7, 6.7)	5.9 (5.5, 6.5)	0.13

Data presented as median (interquartile range, IQR) were compared using the Mann–Whitney U test.

Abbreviations: VAS(R), Visual Analog Scale at rest; VAS(M), Visual Analog Scale on movement.

© 2022 Qiu D et al. *JAMA Network Open.*

eTable 3. Postoperative Complications on POD 1 and 3

	Control Group, (n=91)	Esketamine Group, (n=92)	*P*
**POD 1**			
Nausea and vomiting, n (%)	27 (29.7)	34 (37.0)	0.32
Dizziness, n (%)	48 (52.7)	51 (55.4)	0.78
Itching, n (%)	1 (0)	0 (0)	1
Nightmare, n (%)	0 (0)	1 (0)	1
**POD 3**			
Nausea and vomiting, n (%)	5 (5.5)	5 (5.4)	1
Dizziness, n (%)	12 (13.2)	9 (9.8)	0.50
Itching, n (%)	0 (0)	0 (0)	1
Nightmare, n (%)	0 (0)	0 (0)	1

Data presented as the number of patients (%) were compared using the Pearson chi-square test or Fisher exact test.

Abbreviations: POD 1, postoperative day 1; POD 3, postoperative day 3.

© 2022 Qiu D et al. *JAMA Network Open.*
