# Supplementary material for: Effect of Intraoperative Esketamine Infusion on Postoperative Sleep Disturbance After Gynecological Laparoscopy: A Randomized Clinical Trial
Source: JAMA Netw Open. 2022 Dec 1;5(12):e2244514. doi: 10.1001/jamanetworkopen.2022.44514 (PMC9716381; doi:10.1001/jamanetworkopen.2022.44514)
Supplement: Supplement 3. — Data Sharing Statement [file jamanetwopen-e2244514-s003.pdf]

# Data Sharing Statement

Qiu. Effect of Intraoperative Esketamine Infusion on Postoperative Sleep Disturbance After Gynecological Laparoscopy. *JAMA Netw Open*. Published December 01, 2022. doi:10.1001/jamanetworkopen.2022.44514

**Data**
**Data available:** No
